# Cellular and molecular aberrations generating new immunotherapeutic approaches in mycosis fungoides and Sézary syndrome: a comprehensive review of literature

**DOI:** 10.3389/fimmu.2026.1899436

**Published:** 2026-07-22

**Authors:** Anna Sophia Flechtenmacher, Susanne Melchers, Jan Peter Nicolay

**Affiliations:** 1 Department of Dermatology, Venerology and Allergology, University Medical Center Mannheim/University of Heidelberg, Mannheim, Germany; 2 Section of Immune Dermatology, Allergy and Cutaneous Lymphoma, Medical Faculty Mannheim, University of Heidelberg, Mannheim, Germany; 3 Skin Cancer Unit, German Cancer Research Center (DKFZ), Heidelberg, Germany; 4 DKFZ-Hector Cancer Institute at the University Medical Center Mannheim, Mannheim, Germany

**Keywords:** antibody-drug conjugate (ADC), chimeric antigen receptor (CAR T), CTCL (cutaneous T-cell lymphoma), immunotherapy, molecular target, mycosis fundgoides, Sezary syndrome, tumor microenvironment - TME

## Abstract

Recent advances in molecular and immunologic profiling have substantially refined the understanding of mycosis fungoides (MF) and Sézary syndrome (SS), the two most prominent subtypes of cutaneous T-cell lymphomas (CTCL). CTCL comprise a heterogeneous group of lymphoid malignancies characterized by clonal proliferation of malignant T-cell with cutaneous tropism. The pathogenesis of MF and SS appears to be driven by convergent oncogenic programs involving dysregulated JAK/STAT, NF-κB, PI3K/AKT/mTOR, and MAPK signaling, epigenetic reprogramming, apoptosis resistance, immune escape, and microenvironmental support. In parallel, altered surface phenotypes and chemokine receptor programs shape tissue tropism across skin, blood, and lymph nodes, while the tumor microenvironment promotes tumor persistence and Th2-skewed immune polarization. These insights have translated into novel targeted and immune-based therapies. This review summarizes current insights into the cell-intrinsic and microenvironmental biology of MF and SS and discusses emerging approaches aimed at achieving more durable and personalized disease control.

## 1 Introduction

Cutaneous T-cell lymphomas (CTCLs) are a rare and heterogeneous group of lymphoid malignancies characterized by clonal proliferation of malignant T cells with cutaneous tropism. Mycosis fungoides (MF) is the most common subtype and originates from skin-resident effector memory T-cells (TRM), whereas Sézary syndrome (SS) represents a leukemic and clinically more aggressive variant and arises from central memory T-cells (TCM) with skin-homing capacity (1). Although CTCL includes additional rare entities, such as subcutaneous panniculitis-like T-cell lymphoma (SPTCL) and primary cutaneous aggressive epidermotropic CD8^+^ cytotoxic T-cell lymphoma, this review focuses on MF and SS.

Recent advances in genomic, epigenetic, and immunologic profiling have significantly refined the understanding of MF and SS pathogenesis. Rather than being explained by a single defining lesion, MF and SS appear to arise from heterogeneous but convergent biologic programs involving aberrant transcriptional and epigenetic regulation, dysregulated signaling, apoptosis resistance, immune escape, and microenvironmental support. These processes are further shaped by tissue tropism across skin, blood, and lymph nodes.

The introduction of targeted immunotherapies, such as brentuximab vedotin and mogamulizumab, has significantly improved clinical outcomes in relapsed or refractory MF and SS (1). However, advanced stage MF and SS remain largely incurable outside selected cases undergoing allogeneic hematopoietic stem cell transplantation.

In this review, we provide a comprehensive overview of MF and SS immunobiology, integrating molecular mechanisms, tumor-microenvironment interactions, and emerging therapeutic strategies. We follow a structured approach that progresses from nuclear and intracellular alterations to broader tumor-microenvironment dynamics, highlighting advances and future directions for more precise and durable treatments in MF and SS.

## 2 Cell-intrinsic biology of malignant T-cells

**Figure 1:**
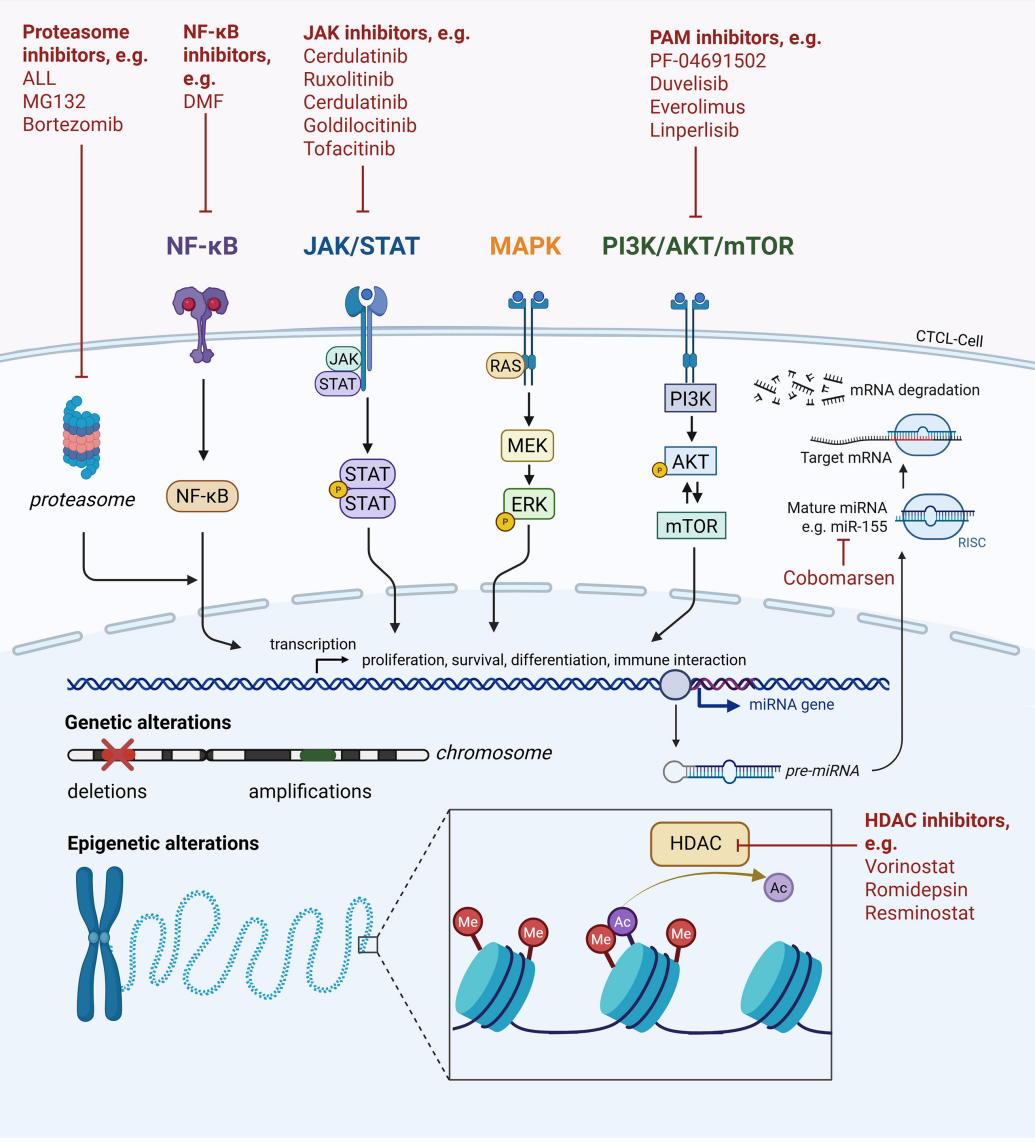
Overview of genetic and epigenetic alteration, intracellular signaling pathways and and therapeutic targets in CTCL. Genetic alterations and epigenetic mechanisms contribute to the activation of key intracellular signaling pathways in Ccells. The NF-κB, JAK/STAT, MAPK, and PI3K/AKT/mTOR pathways promote transcriptional programs involved in proliferation, survival, differentiation, and immune interactions. Examples of therapeutic agents targeting these pathways and epigenetic regulators are shown. Inhibition of miR-155 by cobomarsen represents an additional strategy targeting post-transcriptional gene regulation. Created in https://BioRender.com.

The accumulation of genetic and epigenetic alterations has been proposed to promote a persistent, immune-evasive T-cell phenotype in CTCL (2). Copy-number alterations (CNAs) represent a major class of genomic events, particularly in SS (3). These alterations contribute to the dysregulation of key intracellular signaling pathways, including NF-κB, JAK/STAT, MAPK, PI3K/AKT/mTOR, and T-cell receptor (TCR) signaling, thereby promoting malignant T-cell survival, proliferation, and disease progression. An overview of major cell-intrinsic molecular targets, associated therapeutic approaches, and their current stage of clinical development is provided in **Table 1**. **Figure 1** illustrates genetic and epigenetic alterations, dysregulated signaling pathways, and therapeutic targets in MF and SS.

**Table 1:** Cell-Intrinsic Therapeutic Targets in Mycosis Fungoides (MF) and Sézary Syndrome (SS).

Target	Therapeutic modality	Representative agent(s)	Development status (CTCL)	Major limitations
Epigenetic dysregulation				
HDACs (Class I/II)	HDAC inhibition	Vorinostat (oral), Romidepsin (i.v.) Resminostat	FDA-approved RESMAIN study (Phase II)	Limited durability, toxicity
miR-155	Antisense oligonucleotide	Cobomarsen	Phase I/II	Development discontinued; limited single-agent efficacy
JAK/STAT signaling				
SYK/JAK	Dual inhibition	Cerdulatinib	Phase II (PTCL; includes CTCL subtypes)	Limited CTCL-specific data
JAK1/JAK2	Selective inhibition	Ruxolitinib	Phase II (biomarker-driven)	Modest MF/SS efficacy, risk of CTCL relapse/emergence during therapy
JAK1	Selective inhibition	Golidocitinib	Phase II (PTCL)	Limited CTCL specific data
Pan-JAK	Broad inhibition	Tofacitinib	Case Reports	No prospective CTCL studies
NF-κB signaling				
Proteasome/NF-κB	Proteasome inhibition (NF-κB suppression)	Bortezomib	Preclinical (CTCL-specific)	No dedicated CTCL clinical trials
NF-κB	Direct inhibition	Dimethyl fumarate	Phase II	Gastrointestinal adverse events
PI3K/AKT/mTOR signaling				
PI3K-δ/γ	Dual inhibition	Duvelisib	Phase I	Immune-mediated toxicity
PI3K-δ + HDAC	Combination therapy	Linperlisib + Chidamide	Phase I (CTCL-specific)	Small study size
PI3K-δ/γ + HDAC	Combination therapy	Duvelisib + Romidepsin	Phase Ib/2a (TCL; includes CTCL)	Limited CTCL subgroup data
mTORC1	mTOR inhibition	Everolimus	Phase II (TCL; includes CTCL)	Limited CTCL subgroup data
Dual PI3K/mTOR	Dual inhibition	PF-04691502	Preclinical	No clinical validation
MAPK signaling				
RAS/RAF/MEK/ERK	MEK inhibition	Selumetinib, PD0325901	Preclinical	Rare actionable mutations
TCR Signaling				
ITK/NF-κB/GATA3	ITK inhibition	CPI-818, Ibrutinib	Phase I (CPI-818); Pivotal trial (ibrutinib)	Limited efficacy data
c-CBL/MALT1	MALT1 inhibition	MI-2	Preclinical	No clinical studies
Clonotypic TCR (TRBC/TRBV)	CAR-T, ADCs, bispecific antibodies, vaccines	AUTO4 (TRBC1-CAR T), TRBC-ADCs, TCRVβ-ADCs	Phase I/II (AUTO4); Preclinical (ADCs, vaccines)	Fratricide, T-cell aplasia, patient-specific targeting

Overview of key cell-intrinsic molecular therapeutic targets, current stage of clinical development, and major limitations in Mycosis fungoides (MF) and Sézary Syndrome (SS). ADC, antibody–drug conjugate; CAR-T, chimeric antigen receptor T-cell therapy; CTCL, cutaneous T-cell lymphoma; FDA, U.S. Food and Drug Administration; HDAC, histone deacetylase; PTCL, peripheral T-cell lymphoma; TCR, T-cell receptor.

### 2.1 Genetic and epigenetic drivers of malignant T-cell biology

#### 2.1.1 Genetic alterations

Alterations of the tumor suppressor gene TP53 are among the most frequently reported genomic events in SS (4). Beyond this, the disease has been characterized by recurrent lesions affecting both oncogenes (e.g., *MYC, TOX, STAT3, STAT5B, JAK3*) and tumor suppressor genes (e.g., *RB1, PTEN, CDKN2A, ZEB1*) (5, 6). CNAs commonly included gains of *MYC*, *STAT3/STAT5*, as well as losses of negative regulators such as *MXI1* and *MNT* (7).

In MF, the mutational landscape appears to partially overlap with SS but also showed distinct features. *FAT1* has been described as the most frequently altered gene (~28%), followed by recurrent alterations in *KMT2D, TP53, JAK3*, and *SETBP1* (8). Deletions affecting negative regulators of JAK–STAT signaling (e.g. *CDKN2A, SOCS1*, and *HNRNPK*) may occur even in early-stage disease (9). Alterations in *TNFRSF1B* and *JAK2* fusions, have been shown particularly in more aggressive subtypes (10, 11).

Overall, despite marked inter- and intrapatient heterogeneity, these alterations appear to converge on core pathways, including T-cell receptor signaling, JAK/STAT and NF-κB activation, epigenetic regulation, DNA damage response, and cell-cycle control.

#### 2.1.2 Epigenetic alterations

MF and SS have been further characterized by alterations in epigenetic regulators involved in chromatin remodeling and DNA methylation (including *TET2, DNMT3A, CREBBP, KMT2D/KMT2C, ARID1A, SMARCA4*, and *CHD3)*, leading to a possible disruption of transcriptional control (5, 12).

The reversibility of epigenetic modifications has provided a rationale for therapeutic targeting. Histone deacetylase inhibitors (HDACis), such as vorinostat and romidepsin, are successfully used in advanced CTCL (13). In the phase II RESMAIN trial, the HDACi resminostat prolonged progression-free survival in MF/SS, although toxicity and lack of improvement in patient-reported outcomes highlight limitations of this approach (14). Several next-generation HDACis are under investigation (15).

Preclinical data suggests that HDAC inhibition may enhance transcriptional differences between malignant and normal T cells, potentially improving immune recognition and supporting combination approaches with immunomodulatory therapies (16).

MicroRNAs represent an additional regulatory layer. In SS, upregulation of the oncogenic *miR-155* and downregulation of tumor-suppressive have been reported and linked to key signaling pathways (17). Targeting *miR-155* has shown antitumor activity in preclinical models, but early clinical studies have yielded mixed results (18).

### 2.2 Dysregulated signaling networks

As described above, recurrent alterations in MF and SS converge on a limited number of signaling pathways that regulate proliferation, survival, differentiation, and immune interactions. Key pathways include JAK/STAT, NF-κB, PI3K/AKT/mTOR (PAM), and MAPK, which are now discussed in more detail.

#### 2.2.1 JAK/STAT signaling

Januskinase/Signal Transductor and Activator of Transcription (JAK/STAT) signaling mediates cytokine receptor responses and has been shown to be frequently dysregulated in MF and SS through genetic and epigenetic alterations affecting key pathway components, including JAK1, JAK3, STAT3, and STAT5 (12). This may result in constitutive pathway activation associated with enhanced malignant T-cell proliferation, survival, and resistance to apoptosis. JAK/STAT dysregulation further appears to contribute to a Th1to Th2 phenotype shift, facilitating immune evasion (19, 20).

Pharmacologic JAK inhibition can suppress STAT activation, reduce proliferation, and induce apoptosis in preclinical models (21–23). In a clinical phase II trial, the dual SYK/JAK inhibitor cerdulatinib achieved an overall response rate (ORR) of 35% in relapsed or refractory CTCL, with higher responses in MF (24). Clinical activity has also been reported for ruxolitinib, a selective JAK1/JAK2 inhibitor. However, response rates were lower compared to cerdulatinib, suggesting potential efficacy differences depending on the extent of JAK inhibition and underlying disease biology (25). The selective JAK1 inhibitor golidocitinib showed promising activity in T-cell lymphomas, with an ORR of 44.3% in a phase II trial (26). The pan-JAK inhibitor tofacitinib is being evaluated as a topical therapy in early-stage CTCL (NCT06698822).

Components of the JAK/STAT pathway may also have diagnostic and prognostic relevance. Loss of STAT4 expression has been associated with disease progression (19, 20), whereas increased STAT3 activity has been linked to more aggressive disease (27). Phosphorylated S6 (pS6) has been proposed as a potential biomarker for response to JAK inhibition, although further validation is needed (25).

#### 2.2.2 NF-κB signaling

Nuclear factor-kappaB (NF-κB) regulates immune and survival-related gene expression and plays key roles in T-cell activation, differentiation, and survival (28). In MF and SS, NF-κB appears to be constitutively activated, leading to malignant T-cell survival and resistance to apoptosis (29). In addition, NF-κB activation has been linked to the Th2-skewed cytokine profile characteristic of advanced MF and especially SS, marked by increased expression of IL-4, IL-10, and IL-13 (30).

In preclinical studies NF-κB inhibition reduced NF-κB transcriptional activity and induced apoptosis (29). Proteasome inhibitors such as ALLN, MG132, and bortezomib suppressed NF-κB signaling and triggered apoptotic cell death, with bortezomib indirectly inhibiting NF-κB by preventing its activation and nuclear translocation (29, 31). In clinical studies, bortezomib demonstrated meaningful activity in pretreated CTCL, both as monotherapy and in combination with dexamethasone (NCT03487133), with manageable toxicity (32). Further approaches are under investigation.

Dimethyl fumarate (DMF) has been shown to inhibit NF-κB activity and induce cell death in patient derived SS cells while sparing healthy T cells (33). In a clinical phase II trial, DMF showed clinically meaningful activity in relapsed/refractory CTCL, particularly in SS and high tumor burden, alongside a favorable tolerability profile (34).

Emerging evidence suggests that NF-κB inhibition may contribute to overcoming HDAC inhibitors, supporting combination strategies (35).

#### 2.2.3 PAM signaling

The phosphoinositide 3-kinase (PI3K)/protein kinase B (AKT)/mammalian target of rapamycin (mTOR) (PAM) pathway regulates cellular growth, metabolism, and survival and appears frequently activated in MF and SS, driven by both intrinsic alterations and microenvironmental signals (36, 37). Notably, Sézary cells derived from skin lesions have been shown to exhibit stronger PAM activation and higher proliferative capacity than circulating cells, underscoring the influence of the local microenvironment (37).

In preclinical studies dual PI3K/mTOR inhibitors (e.g. PF-04691502) demonstrated potent growth inhibition, induction of apoptosis and showed greater antitumor activity than mTOR inhibition alone (38).

Clinically, the PI3K-δ/γ inhibitor duvelisib achieved an ORR of 31.6% in CTCL in a phase I trial, while tenalisib and the mTORC1 inhibitor everolimus have shown activity across T-cell lymphomas (39) (40, 41). Combination strategies are explored to enhance efficacy, with encouraging response rates reported for regimens such as linperlisib plus chidamide (ORR 59.1%) and duvelisib plus romidepsin (ORR 55%).

#### 2.2.4 MAP kinase signaling

The mitogen-activated protein kinase (MAPK) pathway represents another central intracellular signaling cascade regulating proliferation, differentiation, survival, and stress responses, classically activated via the RAS–RAF–MEK–ERK cascade downstream receptor signaling.

Aberrant MAPK signaling is increasingly recognized in MF and SS pathogenesis. Mutations in pathway components (e.g., KRAS, NRAS) are relatively rare but appear enriched in advanced or transformed disease, where they have been associated with decreased overall survival (42).

MEK inhibitors have shown selective growth inhibition and apoptosis induction in cell lines harboring KRAS or NRAS mutations (42). The combination of pan-kinase inhibitors showed synergistic effects in CTCL cell lines (43). The MAPK isoform p38γ has been reported to be selectively overexpressed in both MF- and SS-derived cell lines and patient samples, with targeted inhibition showing antitumor activity in preclinical systems (44, 45).

MEK inhibitors have not yet been evaluated in dedicated CTCL trials. Their utility may be constrained by the relatively low frequency of actionable MAPK mutations and potential immunosuppressive effects on T-cell function.

### 2.3 T-cell receptor dysregulation

Malignant T cells in MF and SS retain a functional T-cell receptor (TCR) (46, 47). However, TCR signaling in MF and SS appears to be dysregulated, contributing to tumor cell survival, proliferation, and immune evasion. A characteristic feature is the coexistence of impaired proximal signaling with constitutive activation of downstream pathways. This insight has led to novel therapeutic approaches aimed at either restoring normal TCR signaling to induce activation-induced cell death (AICD) or targeting downstream survival pathways.

#### 2.3.1 Defective proximal TCR signaling

Impaired proximal TCR signaling in MF and SS cells has been characterized by reduced phospholipase Cγ1 (PLCγ1) activation following TCR stimulation, leading to impaired calcium mobilization and decreased reactive oxygen species (ROS) production (48). This has been associated with diminished activity of proximal kinases (e.g. ZAP70, Syk, and Csk) and disrupted coupling of the TCR complex to downstream pathways (48, 49). As a driving mechanism the overexpression of the E3 ubiquitin ligase c-CBL, has been proposed (50).

Functionally, weakened proximal signaling may provide a survival advantage for malignant cells. Normally, repeated TCR stimulation can induce AICD through CD95 ligand (FasL) upregulation. In MF and SS, impaired calcium and ROS signaling limits FasL upregulation and thereby prevents AICD (48). Accordingly, c-CBL inhibition has emerged as a potential therapeutic strategy (50). Experimental studies have shown that c-CBL inhibition restored PLCγ1 phosphorylation, calcium influx, ROS generation, and FasL upregulation, thereby reactivating extrinsic apoptosis pathways (50).

#### 2.3.2 Constitutive activation of downstream signaling

Despite impaired proximal signaling, several downstream pathways have been shown to remain active in MF and SS, partly driven by activating mutations in TCR-associated molecules (2, 51).

One key pathway involves the PLCγ1–PKCθ axis, which can activate STAT3 and promote proliferation (52). Inhibition of PKCθ has been shown to reduce proliferation and induce apoptosis in MF and SS cells (52).

Aberrant TCR signaling may further activate the chemokine receptor CXCR4, leading to stabilization of cytokine transcripts (e.g. IL-2, IL-4, and IL-10), promoting a tumor-supportive Th2-skewed microenvironment (53). Accordingly, multiple CXCR4-targeted approaches are under investigation (54–56). Nevertheless, evidence in MF and SS remains largely preclinical or derives from small exploratory studies, and the clinical utility of CXCR4-directed therapies in CTCL has yet to be established.

Another relevant pathway is the ITK/NF-κB/GATA-3 signaling axis, which has been linked to chemotherapy resistance in T-cell lymphomas (57). While ITK inhibition has been shown to restore chemosensitivity in preclinical models, clinical responses to ibrutinib have been limited (57, 58) (59).Newer strategies, including ITK degraders and selective inhibitors such as CPI-818, are currently being explored (60).

#### 2.3.3 TCR clonotypes

In contrast to the diverse T-cell receptor (TCR) repertoire of normal T cells, both MF and SS are characterized by a restricted TCR repertoire (61). Certain TCR variable segments (e.g., Vβ20, Vγ8) have been linked to more aggressive disease but lack tumor specificity, limiting therapeutic utility due to the risk of broad T-cell depletion (47). In contrast, the full clonotypic TCR sequence, particularly the highly variable CDR3 region, is unique to the malignant clone and represents a patient- and tumor-specific target.

Preclinical data suggest that clonotypic TCR sequences can be processed and presented via major histocompatibility complex (MHC) class I molecules, eliciting tumor-specific immune responses and supporting their potential as immunogenic targets (62–64).

Multiple targeting strategies are under investigation, including CAR T-cells directed against defined TCR variable β chains (TCRVβ), CAR-engineered invariant natural killer T (iNKT) cells, bispecific antibodies targeting TRBV regions, and antibody–drug conjugates (ADCs) targeting TRBC1/2 (65) (66, 67). Notably, TRBC-directed ADCs have demonstrated selective cytotoxicity in preclinical models and may circumvent limitations of cellular therapies, such as fratricide (67, 68). DNA vaccine approaches targeting TCR chains have been shown to induce immune responses and delay tumor progression in preclinical models (69, 70).

## 3 Surface phenotype of malignant T cells

**Figure 2:**
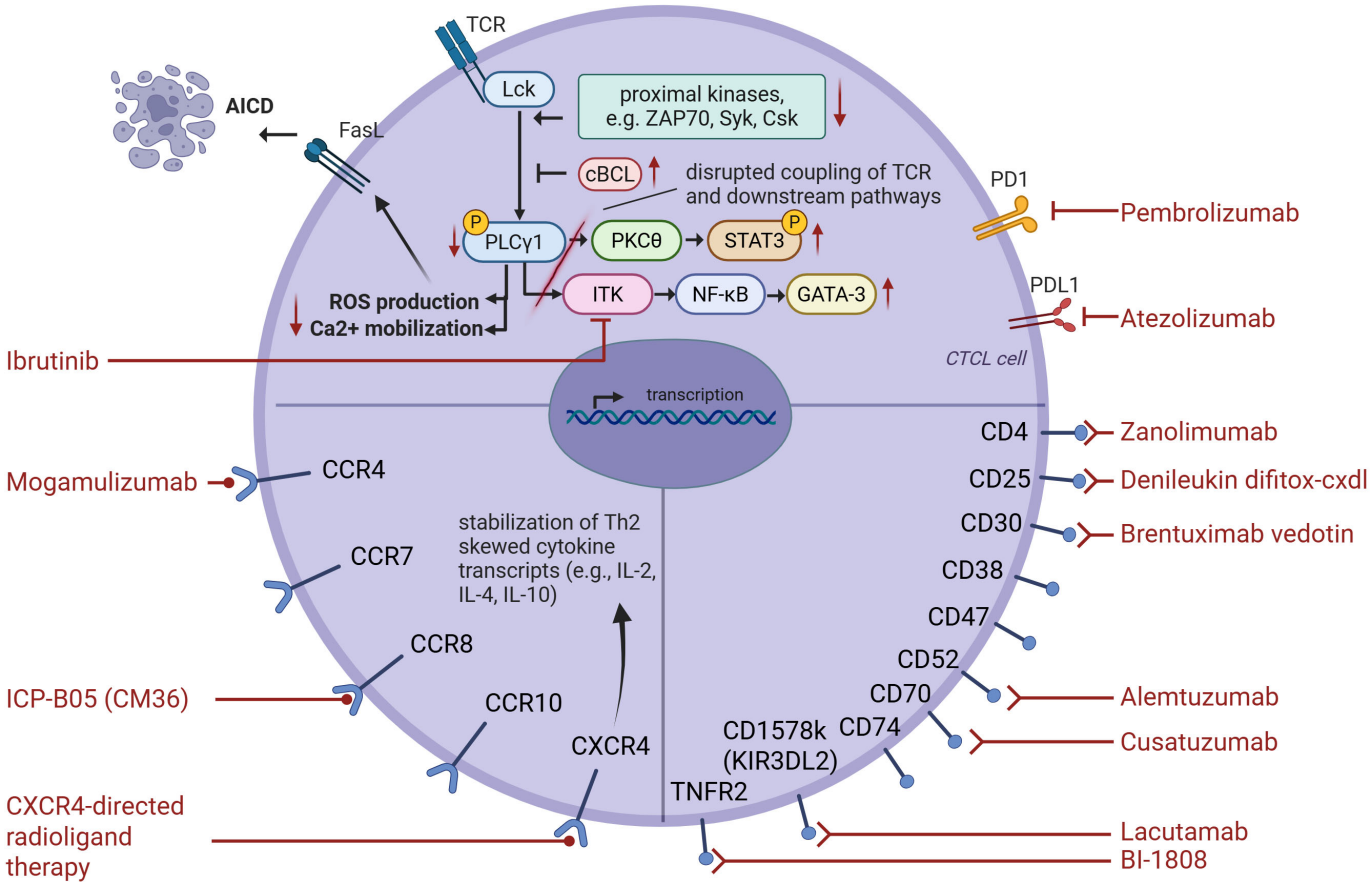
Dysregulated T-cell receptor (TCR) signaling, surface receptors, and therapeutic targets in CTCL. Shown are key abnormalities of TCR signaling and therapeutically relevant surface receptors in CTCL cells. Therapeutically relevant surface molecules comprise chemokine receptors involved in tissue tropism (CCR4, CCR7, CCR8, CCR10, and CXCR4), immune checkpoint molecules (PD-1/PD-L1), and targetable CD antigens and receptors. Examples of corresponding therapeutic agents are indicated. Created in https://BioRender.com.

Malignant T cells in MF and SS exhibit an aberrant surface immunophenotype characterized by altered expression of cluster of differentiation (CD) molecules and receptors. These changes likely reflect underlying genetic and epigenetic alterations and contribute to dysregulated signaling, immune evasion, and tissue tropism (1, 71). An overview of therapeutically relevant cell-surface molecules, their clinical development status, and associated limitations is provided in **Table 2**, while **Figure 2** illustrates therapeutically relevant surface receptors.

**Table 2:** Cluster of Differentiation (CD) Molecules and Related Surface Proteins as Therapeutic Targets in Mycosis Fungoides (MF) and Sézary Syndrome (SS).

Target	Expression pattern in MF/SS	Therapeutic modality	Development status (CTCL)	Major Limitations
CD4	Universally expressed on MF/SS cells	Monoclonal Antibody (zanolimumab)	Phase II	Profound immune-suppression
CD25 (IL-2Rα)	Variable, context-dependent expression in MF/SS	Fusion protein (denileukin diftitox-cxdl)	Phase III / FDA approved (R/R Stage I–III CTCL)	Capillary leak syndrome
CD30	Variable expression; higher in LCT and tumor stage MF	ADC (brentuximab vedotin); CAR T-cell therapies	Brentuximab vedotin: Phase III / FDA approved (CD30+ MF after ≥1 prior therapy) CAR T: Preclinical /early clinical	Heterogenous expression; Peripheral neuropathy; CAR T: fratricide, T-cell aplasia, malignant-cell contamination, CRS, ICANS
CD38	Variable expression in MF, often low in SS	Monoclonal antibodies (e.g. daratumumab)	Preclinical (CTCL)	Heterogenous expression; loss of CD38; off-tumor effects
CD47	Frequently overexpressed on MF and SS cells	Fusion protein (ontorpacept)	Phase I	"antigen sink"; off-tumor effects; hematological toxicity
CD52	Broad expression on malignant and normal lymphocytes	Monoclonal antibody (alemtuzumab)	Phase II	Severe immune-suppression
CD70	transiently expressed on activated lymphocytes, more consistently on MF and SS cells	Monoclonal antibody (Cusatuzumab); Allogeneic CAR-T (CTX130); ADC (SGN-CD70A)	Phase I	See above challenges of CAR-T therapies; off-tumor toxicity; anti-drug antibody formation
CD74	Broadly and robustly expressed on MF and SS cells	ADC (STRO-001)	Preclinical	CD74 broadly expressed on B cells, macrophages, dendritic cells → potential off-tumor effects
CD158k (KIR3DL2)	High in SS, subset of MF	Monoclonal Antibody (lacutamab)	Phase II (TELLOMAK); Breakthrough Therapy Designation (Feb 2025)	Heterogeneous expression in MF; variable compartment responses; physiological expression on NK/CD8+ T cells
TNFR2	Expressed on MF, SS cells Tregs and activated T cells	TNFR2 antagonist (BI-1808)	Phase I/IIa	Limited efficacy data

Overview of major cell-surface molecules and their potential therapeutic targeting in mycosis fungoides (MF) and Sézary syndrome (SS). ADC, antibody–drug conjugate; CAR-T, chimeric antigen receptor T-cell therapy; CRS, cytokine release syndrome; CTCL, cutaneous T-cell lymphoma; FDA, U.S. Food and Drug Administration; ICANS, immune effector cell-associated neurotoxicity syndrome; LCT, large-cell transformation; NK, natural killer; R/R, relapsed/refractory; Treg, regulatory T cell.

### 3.1 Diagnostic and biologic marker patterns

A frequently observed feature of CTCL is the partial loss of pan-T-cell markers such as CD7 and CD26, which is widely used diagnostically in histology and flow cytometry (1, 71). Additional markers may provide biological and prognostic insights, although their expression can be variable.

### 3.2 Functionally relevant therapeutic surface targets

A subset of surface markers in MF and SS has been identified as functionally involved in disease biology and therapeutically targetable.

CD4 is characteristically expressed on malignant T-cells in MF and SS. The anti-CD4 monoclonal antibody zanolimumab has demonstrated clinical responses in MF and SS in early-phase trials (72, 73). However, because CD4 is also broadly expressed on the majority of normal helper T cells, therapeutic targeting inevitably results in depletion of non-malignant CD4+ T cells and may therefore cause clinically relevant immunosuppression limiting the therapeutic window (73). Although zanolimumab showed an acceptable safety profile in early clinical studies, its further clinical development was discontinued and it did not achieve regulatory approval (72).

CD25 (IL-2Rα) may support IL-2-dependent signaling and contribute to immune modulation, although its expression is heterogeneous and context-dependent (74). Targeting CD25 with denileukin diftitox-cxdl has demonstrated clinical activity and has been approved by the FDA for relapsed or refractory stage I–III CTCL after prior systemic therapy, although toxicity (e.g., capillary leak) remains a concern (75).

CD30 has been shown to be variably expressed in transformed MF, potentially promoting tumor cell survival, proliferation, and resistance to apoptosis (e.g., by activation of NF-κB) (76, 77). Clinically, CD30 represents an established therapeutic target, with the fusion toxin brentuximab vedotin showing significant efficacy in CD30-positive CTCL (78). Combination strategies are under evaluation. In addition, CD30-directed cellular therapies, including CAR T-cell approaches, present a promising but still investigational strategy. Compared with pan-T-cell targets, CD30 offers the advantage of restricted expression on normal resting T cells, potentially reducing on-target off-tumor toxicity. However, heterogeneous and often variable CD30 expression in MF and SS may limit therapeutic efficacy and patient eligibility. Furthermore, as with other T-cell-directed immunotherapies in t-cell lymphomas, challenges such as fratricide, T-cell aplasia, contamination of the product with malignant T cells and the lack of truly tumor-specific antigens must be considered (79).

CD38 has demonstrated heterogeneous and stage-dependent expression in MF (80, 81). It may modulate proliferation, migration, and metabolic fitness, while also contributing to an immunosuppressive tumor microenvironment (82). Anti-CD38 antibodies such as daratumumab have shown antitumor activity in preclinical MF models; however, heterogeneous expression, loss of CD38 expression in residual tumor cells, and high expression of complement inhibitory proteins may limit therapeutic efficacy (82). Clinical studies of CD38-directed therapies in T-cell lymphomas have demonstrated limited efficacy and significant toxicity (83, 84). Combination approaches (e.g., with HDAC inhibitors) are being explored. By contrast, Sézary cells appear to exhibit a predominantly CD38-negative phenotype, potentially limiting CD38-targeted therapies in SS (80).

CD47 has been shown to be frequently overexpressed on MF and SS cells and may contribute to immune evasion by inhibiting phagocytosis via SIRPα engagement (“don’t eat me signal”) (85, 86). Therapeutic targeting of this axis is discussed in the context of immune escape below.

CD52 has been detected in most MF and SS cases, although expression levels seem to vary (87). The anti-CD52 antibody alemtuzumab has shown clinical activity in CTCL, particularly in SS and erythrodermic disease (88). However, its clinical applicability is limited by profound immunosuppression associated with high risk of opportunistic infections, requiring antimicrobial prophylaxis and close monitoring (89). Current guidelines suggest the use of alemtuzumab only in selected advanced cases (90, 91).

CD70 is transiently expressed on activated lymphocytes but appears to be more consistently expressed in MF and SS, potentially contributing to immune evasion, tumor cell proliferation and survival (92). Early clinical studies with the anti-CD70 antibody cusatuzumab demonstrated modest activity, with higher response rates reported in SS (93). However, anti-drug antibody formation limited drug exposure and may have contributed to loss of response in some patients (93). CD70-directed CAR T-cell therapy has shown encouraging clinical activity in CTCL, while antibody-drug conjugates targeting CD70 demonstrated potent antitumor effects in preclinical MF models (92–94). Nevertheless, the clinical applicability of CD70-targeting therapies remains limited and no CD70-directed therapy has yet achieved regulatory approval for CTCL. Next-generation constructs (CTX131) and combination strategies are under investigation to address these limitations.

CD74 is involved in antigen presentation and primarily expressed on B cells. Its consistent expression on malignant T cells in MF and SS is notable and has been linked to pro-survival pathways (95). Antibody–drug conjugates (ADCs) directed against CD74, such as STRO-001, have shown promising preclinical activity in CTCL models (95). However, evidence for CD75 targeting in CTCL remains preclinical. The broad expression of CD74 on normal immune cells raises concerns regarding on-target, off-tumor toxicity, while rapid CD74 internalization and turnover may limit therapeutic exposure (96). Although ADC-based approaches may partially exploit these biological properties to enhance intracellular drug delivery, clinical validation in MF and SS is still lacking.

CD158k (KIR3DL2) is reported to be aberrantly expressed on CTCL cells, particularly in SS and transformed MF and serves as a sensitive diagnostic marker (97). Functionally, it may contribute to immune evasion and tumor cell survival. Higher expression has been associated with more advanced disease stages and poorer outcomes (98). Therapeutically, the anti-KIR3DL2 antibody lacutamab has shown encouraging activity in the TELLOMAK phase II trial, especially in SS, with a favorable safety profile (99). However, physiological expression of KIR3DL2 on normal NK cells and a subset of CD8+ T cells, heterogeneous expression in MF and variable responses across disease compartments may limit the broader applicability of this approach (99, 100). The ongoing phase III TELLOMAK-3 trial will further define its clinical benefit.

TNFR2 (tumor necrosis factor receptor 2) has been proposed to play a dual role in tumor biology. Recurrent mutations and gains may enhance NF-κB signaling promoting malignant T-cell survival (10). In parallel, TNFR2 expressed on suppressive Tregs may contribute to immune evasion (101). Preclinical studies show that TNFR2 antagonism can deplete both malignant cells and Tregs while restoring effector T-cell function (102). Early clinical trials (e.g., of BI-1808) demonstrate favorable safety and preliminary activity (103). However, the clinical utility of TNFR2-directed approaches may be limited by the context-dependent functions of TNFR2 in both regulatory and effector immune cells, the absence of validated predictive biomarkers, and the modest efficacy observed in early clinical studies (103). Ongoing studies are evaluating combination strategies, particularly with PD-1 blockade (103).

## 4 Functional consequences of molecular alterations

Disease progression in MF and SS appears to reflect a combination of increased proliferative activity and multifactorial apoptosis resistance, involving both extrinsic and intrinsic mechanisms. **Table 3** provides an overview of potential therapeutic targets regulating proliferation, apoptosis resistance and survival in MF and SS.

**Table 3:** Therapeutic Targeting of Proliferation, Apoptosis Resistance, and Cytokine-Supported Survival in Mycosis Fungoides (MF) and Sézary Syndrome (SS).

Target	Therapeutic modality	Representative agent(s)	Development status (for CTCL)	Major limitations
Proliferation & cell cycle control				
FOXM1	FOXM1 inhibition	RCM1, FDI-6	Preclinical	No clinical data
Extrinsic apoptosis defects				
Fas/CD95	Fas restoration	Methotrexate, IFN-α	FDA-approved for MF (MTX); NCCN-listed, not FDA-approved for CTCL (IFN-α)	Fas restoration alone insufficient due to multifactorial apoptosis resistance
Extrinsic apoptosis pathway	Apoptosis induction	Gentian violet (GV)	Preclinical	No clinical validation
Intrinsic apoptosis buffering				
BCL-2	BCL-2 inhibition	Venetoclax	Preclinical/ex vivo (CTCL-specific)	No CTCL-specific clinical trial Case report
MCL-1	MCL-1 inhibition	AZD5991	Preclinical	No CTCL-specific clinical data
BCL-2 + HDAC	Combination therapy	Venetoclax + Vorinostat/Romidepsin	Preclinical	No clinical validation
BCL-2 + NF-κB	Combination therapy	Venetoclax + DMF	Preclinical	No clinical validation
BCL-2 + BET	Combination therapy	JQ1, ABBV-075 + Venetoclax	Preclinical	No clinical validation
Cytokine-supported survival				
IL-7	IL-7 blockade	Anti-IL-7 antibody	Preclinical	Risk of immunosuppression
IL-9	Cytokine blockade	BNZ-1	Preclinical	No CTCL-specific clinical trial
IL-10	IL-10 blockade	Experimental (anti-IL-10 antibody)	Preclinical	No therapeutic candidate in development; risk of autoimmunity
IL-15	IL-15 modulation	rhIL-15, BNZ-1	Preclinical (T-LGLL/ATL models, not CTCL-specific)	Limited CTCL-specific data

Overview of therapeutic targets regulating proliferation, apoptosis resistance, and survival in mycosis fungoides (MF) and Sézary syndrome (SS). BET, bromodomain and extraterminal domain; CTCL, cutaneous T-cell lymphoma; DMF, dimethyl fumarate; FDA, U.S. Food and Drug Administration; FOXM1, forkhead box protein M1; GV, gentian violet; HDAC, histone deacetylase; IFN-α, interferon-α; IL, interleukin; MCL-1, myeloid cell leukemia 1; MF, mycosis fungoides; MTX, methotrexate; NCCN, National Comprehensive Cancer Network; NF-κB, nuclear factor kappa-light-chain-enhancer of activated B cells; PTCL, peripheral T-cell lymphoma; R/R, relapsed/refractory; rhIL-15, recombinant human interleukin-15; SS, Sézary syndrome; T-LGLL, T-cell large granular lymphocytic leukemia.

### 4.1 Proliferation and cell cycle control

Proliferative activity increases with disease stage, as indicated by elevated Ki-67 expression in advanced MF (104). Genetic alterations affecting cell-cycle regulators, particularly loss of CDKN2A/CDKN2B, have been associated with aggressive disease and reduced survival (105). Transcriptional drivers such as *FOXM1* have been shown to be linked to poor prognosis in advanced MF (106). Experimental inhibition of FOXM1 reduced proliferation and induced cell-cycle arrest, supporting its potential as a therapeutic target (106).

### 4.2 Apoptosis resistance and survival programs

#### 4.2.1 Extrinsic apoptosis defects

Impaired death receptor signaling represents a recurring feature of apoptosis resistance in MF and SS. Reduced Fas (CD95) expression, impaired receptor signaling, and overexpression of inhibitory regulators such as c-FLIP contribute to resistance to Fas-mediated apoptosis (107–109). CTCL cells show reduced sensitivity to multiple death ligands (e.g., TNF-α, TRAIL), associated with impaired activation of caspase-8 and -3, as well as loss of components such as TNF-R1 and caspase-10 (110). Defective activation-induced cell death (AICD), a key mechanism for eliminating activated T cells via Fas (CD95)-FasL signaling, further contributes to tumor persistence (48).

Therapeutically, strategies aimed at restoring death receptor function and overcoming apoptosis resistance therefore appear promising. Experimental approaches include upregulation of Fas expression (e.g., by epigenetic modulation such as methotrexate) and restoration of upstream TCR signaling, which can re-sensitize CTCL cells to apoptosis (111). Interferon-α has been shown to increase Fas expression, while agents such as gentian violet may enhance Ca²^+^/ROS signaling and FasL induction in preclinical models (111, 112). Given the multifactorial nature of apoptosis resistance in MF and SS, combination strategies are likely required. These may include concurrent enhancement of extrinsic apoptosis signaling (e.g., Fas/FasL) together with targeting of survival pathways (such as NF-κB) or intrinsic apoptosis regulators (e.g., BCL-2 family proteins), which have shown synergistic effects in preclinical systems (33, 113).

#### 4.2.2 Intrinsic apoptosis buffering

Intrinsic apoptosis resistance in MF and SS is proposed to be mediated by dysregulation of the BCL-2 family. Antiapoptotic proteins such as BCL-2, BCL-xL, and MCL-1 have been shown to be commonly expressed, while proapoptotic signaling is functionally impaired (114).

Cytokine signaling, particularly via IL-7 and IL-15, may further reinforce survival through JAK/STAT-mediated upregulation of antiapoptotic proteins (115).

BH3 mimetics such as venetoclax have demonstrated activity in CTCL models, although responses are heterogeneous and likely depend on the dominant antiapoptotic dependency (e.g., BCL-2 vs. MCL-1) (113, 116). Combination strategies, including HDAC inhibitors or targeting JAK/STAT and NF-κB pathways, have demonstrated enhanced efficacy of Bcl-2 inhibitors in preclinical studies (23, 116).

#### 4.2.3 Cytokine-supported survival

Cytokines such as IL-7, IL-15, IL-13, and IL-9 appear contribute to tumor maintenance via autocrine and paracrine signaling mechanisms (115, 117, 118). Therapeutic strategies aim to exploit or modulate these pathways. Recombinant IL-15 (rhIL-15) has shown limited efficacy as monotherapy in early clinical trials across advanced malignancies but induced robust expansion of cytotoxic effector cells, including marked increases in NK cells (119). This provides a rationale for combination approaches in MF and SS, particularly with immune checkpoint inhibitors or antibody-based therapies (120).

## 5 Skin homing and tissue tropism

Compartmentalization of malignant T cells across skin, blood, and lymph nodes in MF and SS has been proposed to be shaped by adhesion molecules and chemokine receptors that regulate microvascular arrest, extravasation and local positioning. **Table 4** provides an overview of important chemokine receptors and their potential therapeutic targeting in MF and SS. **Figure 2** complements this by providing a graphical overview of key therapeutic targets on the malignant T-cell surface.

**Table 4:** Chemokine Receptors as Therapeutic Targets in Mycosis Fungoides (MF) and Sézary Syndrome (SS).

Target	Expression pattern in MF/SS	Therapeutic modality	Clinical evidence	Major limitations
CCR4	Highly expressed in MF/SS	Monoclonal antibody (mogamulizumab)	Phase III / FDA approved (R/R MF/SS after ≥1 prior therapy)	Resistance, immune-mediated toxicity
CCR7	Expressed on circulating Sézary cells	No specific agents in CTCL	Preclinical / conceptual	CCR7 broadly expressed on naive and central memory T cells → high off-target risk
CCR8	Expressed on malignant MF/SS cells and Tregs	Monoclonal antibody (ICP-B05); CAR T-cell therapy	Phase I (ICP-B05 in CTCL; first-in-disease) CAR T (preclinical)	Limited clinical data with limited patient numbers, variable CCR8 expression
CCR10	Expressed in MF/SS skin biopsies and circulating malignant cells in MF/SS	No specific agents	Preclinical / conceptual	CCR10 expressed on normal skin-homing CLA+ T cells → high off-target risk
CXCR4	Expressed on MF cells in early patch/plaque stages; expressed on Sézary cells	Radioligand therapy (^90^Y-Pentixather); Antagonists (mavorixafor, plerixafor)	Preclinical/ Conceptual (No CTCL specific clinical data)	Stage-dependent expression; high off-target risk; CXCR4 antagonism theoretical risk of malignant cell mobilization

Overview of important chemokine receptors and their potential therapeutic targeting in Mycosis fungoides (MF) and Sézary Syndrome (SS). CAR T-cell, chimeric antigen receptor T-cell; CTCL, cutaneous T-cell lymphoma; FDA, U.S. Food and Drug Administration; R/R, relapsed/refractory; Treg, regulatory T cell.

### 5.1 Cutaneous lymphocyte antigen and selectin-mediated vascular capture

Cutaneous lymphocyte antigen (CLA) appears to mediate skin-specific adhesion through interaction with E-selectin on cutaneous endothelium (121). Malignant cells in MF and SS commonly express CLA, consistent with their skin-directed tropism (46). Expression tends to be higher in early epidermotropic disease and may decrease in advanced stages (122). Preclinical studies suggest that targeting CLA or its biosynthesis can impair T-cell trafficking and tumor dissemination (123).

### 5.2 Chemokine receptor programs

CCR4 is highly expressed on malignant T cells in both MF and SS. In MF, CCR4 expression has been shown to increase with disease stage, while in SS, circulating and dermal Sézary cells have been identified as consistently CCR4-positive (124, 125). CCR4 has been shown to play a central role in skin homing through interaction with its ligands CCL17 (TARC) and CCL22 (125). Beyond trafficking, CCR4 signaling may support survival and contribute to immune evasion, partly through recruitment of CCR4^+^ regulatory T cells (126, 127). Therapeutically, CCR4 is targeted by the monoclonal antibody mogamulizumab, which is further described below.

CCR7 mediates lymph node homing and seems to show distinct expression patterns in MF and SS (128). In SS, circulating malignant cells often express CCR7^+^ as part of a central memory T-cell phenotype, enabling trafficking between blood, skin, and lymph nodes, whereas skin-resident MF cells are typically CCR7^-^ (46). CCR7 expression has been associated with systemic dissemination in SS and may promote migration and invasion, partly through PI3K/Akt/mTOR pathways (129). While currently not directly targetable, inhibition of these downstream pathways has shown clinical activity in CTCL (15). PI3K inhibitors (e.g., duvelisib) and mTOR inhibitors may, at least in part, interfere with CCR7-associated signaling programs.

CCR8 has been shown to be expressed both on malignant T cells as well as on tumor-infiltrating regulatory T cells (Tregs) (130). Ligand engagement may activate MAPK signaling and promote tumor survival, while CCR8^+^ Tregs contribute to local immune evasion (131). Early clinical data suggest activity of CCR8-targeting antibodies (e.g. ICP-B05 (CM369)), although development remains at an early stage (130).

CCR10 may contribute to skin homing via interaction with the keratinocyte-derived ligand CCL27 and may promote survival through PI3K/Akt and MAPK pathways (132, 133). Its expression has been documented in a substantial subset of MF and SS cases (134). Targeting CCR10 remains at a preclinical stage.

CXCR4 has been shown to be expressed on malignant T cells in MF and SS, while its ligand CXCL12 is abundant in the skin, promoting direct migration (135). In MF CXCR4 expression has been documented highest in patch and plaque stages and downregulated in tumor-stage disease, suggesting a role in early disease biology rather than advanced progression (136). Beyond migration, CXCR4 signaling may promote survival via PI3K/AKT and contribute to cytokine dysregulation through TCR-CXCR4 crosstalk (53). Preclinical models show that inhibition of the CXCL12/CXR4 axis increased chemosensitivity and reduced MF cell motility (137). Clinically, CXCR4-directed radioligand therapy (^90^Y-Pentixather) has shown encouraging signals in small cohorts with advanced T-cell lymphoma, while CXCR4 antagonists (e.g. plerixafor and mavorixafor) demonstrated antitumor and chemosensitizing activity in hematologic malignancies with CTCL-specific data remaining limited (56, 138).

## 6 Tumor microenvironment and immune crosstalk

Disease progression in MF and SS appears to be associated with an increasingly immunosuppressive microenvironment and a predominance if Th2-dominated immune responses. In MF, a gradual shift from a Th1- to a Th2-dominated immune response has been described. Early-stage lesions have been shown to be relatively enriched in Th1 cytokines (e.g., IFN-γ, IL-2, IL-12), whereas advanced disease has been characterized by increased expression of Th2 cytokines such as IL-4, IL-5, IL-10, and IL-13 (139, 140). In parallel, Th2-associated chemokines (including CCL17, CCL18, CCL22, and CCL26) are upregulated, while Th1-associated chemokines (e.g., CXCL9–11) are reduced (141). SS seems to exhibits a predominantly Th2-skewed cytokine profile in both skin and blood can reduce from disease onset. Experimental models suggest that interruption of IL-10 signaling tumor growth, macrophage infiltration, and M2 polarization (142).

**Figure 3:**
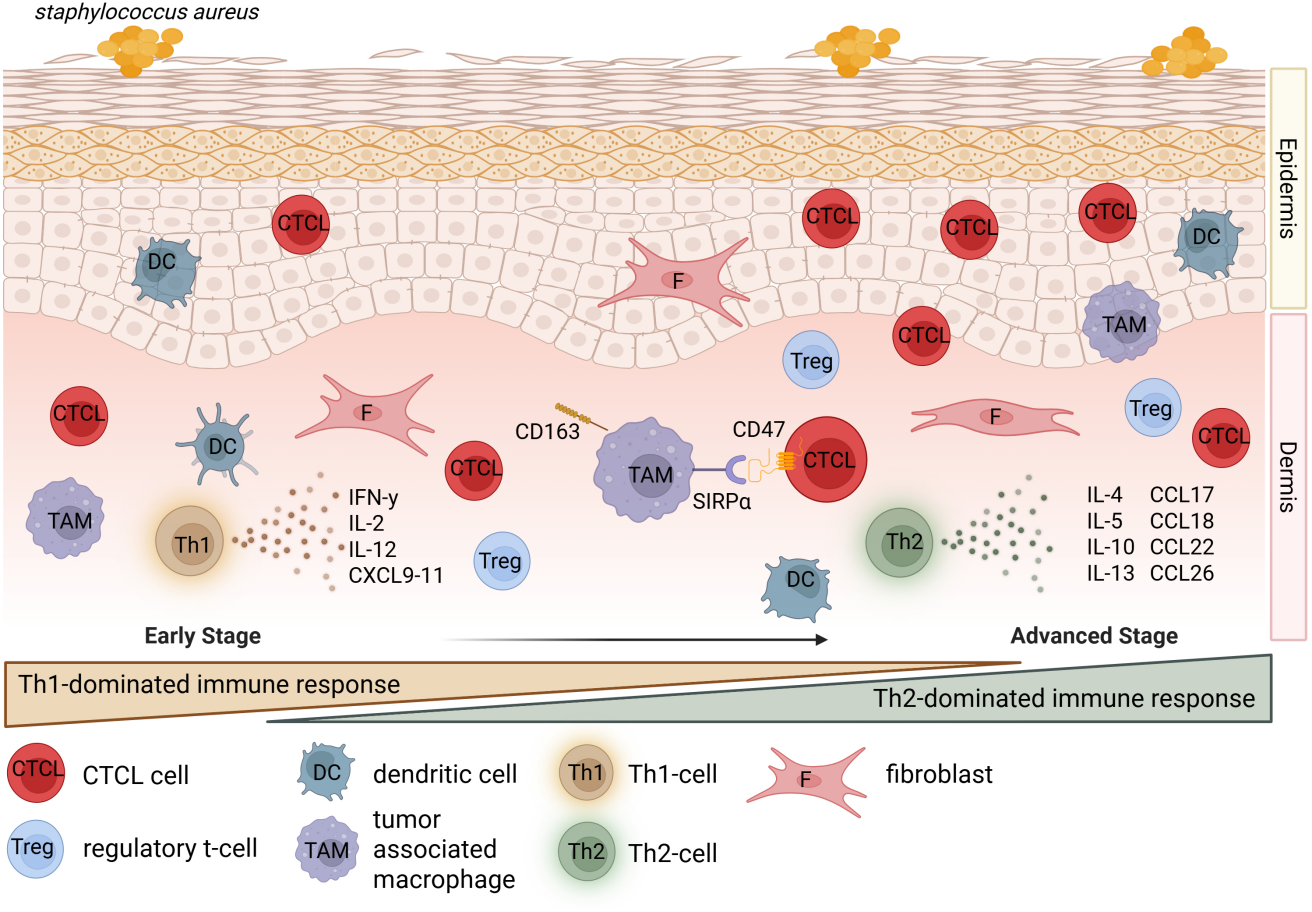
Tumor microenvironment and immune crosstalk in CTCL. The schematic illustrates the transition from a Th1-dominated immune response in early-stage disease to a Th2-dominated and immunosuppressive tumor microenvironment in advanced CTCL. Shown are malignant T cells (CTCL) and their interactions with dendritic cells (DC), tumor-associated macrophages (TAMs), fibroblasts (F), and regulatory T cells (Treg). The image further illistrates CD47-SIRPa-mediated suppression of macrophage phagocytosis and the proposed role of staphylococcus aureus in promoting immune dysregulation and tumor progression. Created in https://BioRender.com.

Functionally, Th2 cytokines, particularly IL-4 and IL-13, can inhibit Th1 differentiation and dampen cytotoxic T-cell responses. This cytokine reprogramming likely contributes to impaired antitumor immunity and may facilitate the survival and expansion of malignant T cells within the skin (140). **Table 5** provides an overview over tumor microenvironment-directed therapeutic strategies in MF and SS, while **Figure 3** illustrates the interactions between malignant T cells and key microenvironmental components, including dendritic cells, tumor-associated macrophages, fibroblasts, regulatory T cells, and *Staphylococcus aureus*.

**Table 5:** Tumor microenvironment–directed therapeutic strategies in Mycosis Fungoides (MF) and Sézary Syndrome (SS).

Target	Therapeutic modality	Repersentative agent(s)	Development status (CTCL)	Major limitations
Th1→Th2 cytokine shift	Th1 restoration / Th2 suppression	IFN-α, IFN-γ-1b	Clinical (NCCN-listed)	Not FDA-approved for CTCL; flu-like symptoms; limited efficacy in advanced disease as monotherapy
M2 TAM polarization (CD163^+^)	TAM modulation	Lenalidomide; Durvalumab + Lenalidomide	Phase II / early clinical	Toxicity; limited cohort size
CCR2 (TAM recruitment)	Macrophage recruitment inhibition	CCR2 inhibitors	Preclinical / early clinical	No CTCL-specific data
DC maturation / antigen presentation	Immunomodulation	Extracorporeal photopheresis (ECP)	FDA-approved / NCCN-listed	mechanism incompletely understood; specialized equipment required; best efficacy in erythrodermic MF/SS
DC-based vaccination	Tumor-lysate DC vaccination	Intranodal DC injection	Phase I/II (CTCL-specific)	Small cohorts; individualized production; no randomized trials
FAP-α^+^ cancer-associated fibroblasts (CAFs)	FAP-directed therapy	Experimental	Preclinical	No clinical validation
CAF-mediated stromal signaling (CXCL12, IL-6/JAK2/STAT3)	Stromal signaling disruption	Experimental	Preclinical	No clinical validation
FOXP3^+^ Treg compartment	Indirect Treg modulation	Checkpoint inhibitors, IFN-α, ECP	Clinical	Uncertain biological role
S. aureus colonization	Antibiotic eradication	Anti-staphylococcal antibiotics	Clinical (supportive care)	Recolonization; uncertain survival benefit
S. aureus superantigens	Disruption of superantigen signaling	Experimental	Preclinical	No targeted therapies available

Overview of therapeutic strategies targeting key components of the tumor microenvironment in mycosis fungoides (MF) and Sézary syndrome (SS). CAF, cancer-associated fibroblast; CTCL, cutaneous T-cell lymphoma; DC, dendritic cell; ECP, extracorporeal photopheresis; FDA, U.S. Food and Drug Administration; IFN, interferon; JAK, Janus kinase; MF, mycosis fungoides; NCCN, National Comprehensive Cancer Network; SIRPα, signal regulatory protein alpha; SS, Sézary syndrome; STAT, signal transducer and activator of transcription; TAM, tumor-associated macrophage; Treg, regulatory T cell.

### 6.1 Tumor-associated macrophages

Tumor-associated macrophages (TAMs) have been identified as a major component of the infiltrate in CTCL lesions. In MF and SS, they seem to be predominantly polarized toward an M2-like phenotype (CD163^+^), particularly in advanced disease. Increased TAM density and higher CD163/CD68 ratios have been associated with poorer prognosis (143). Preclinical studies suggest that TAM depletion reduced tumor growth (144). Single-cell analyses further revealed active crosstalk between malignant T cells and macrophages through pathways such as CD47–SIRPα and S100A9–TLR4 (85).

These findings provide a rationale for macrophage-directed strategies. Among these, blockade of the CD47–SIRPα axis is currently the most advanced and is discussed further below. Additional approaches include combinations with PD-1/PD-L1 blockade, lenalidomide, CCR2 inhibition, and cellular therapies that may indirectly modulate macrophage function (145–147). Lenalidomide is of particular interest in CTCL due to its immunomodulatory effects on both innate and adaptive immunity, including enhancement of T-cell and NK-cell activity and potential modulation of TAM polarization (148). Clinical studies in relapsed or refractory CTCL have demonstrated modest but durable responses, although toxicities such as fatigue, cytopenia, edema, and tumor flare reactions remain limiting factors (149, 150).

### 6.2 Dendritic cells

Malignant T cells in MF and SS appear to impair dendritic cell (DC) maturation and function, promoting tolerance over effective antitumor immune defense (151). Although multiple DC subsets are present in MF and SS lesions, many appear phenotypically immature (152). Experimental models suggest that immature DCs can support malignant T cell survival and growth (153).

These observations have therapeutic implications. Extracorporeal photopheresis (ECP), a standard treatment for erythrodermic MF and SS, has been shown to induce immunogenic cell death and promote differentiation of monocytes into functional DCs (154). Clinical data showed reductions in circulating tumor burden and clinical improvement in a substantial proportion of patients, accompanied by shifts toward a pro-inflammatory cytokine milieu (155). Direct DC-based vaccination using autologous tumor-lysate–pulsed DCs has also shown early clinical activity, particularly in patients with lower tumor burden, and may induce tumor-specific CD8+ T-cell responses (156).

### 6.3 Fibroblasts

In MF and SS cancer-associated fibroblasts (CAFs) may contribute to a tumor-supportive, Th2-skewed microenvironment. Unlike normal fibroblasts, which promote Th1 polarization, MF-derived fibroblasts have been shown to sustain malignant T-cell phenotypes and tumor-supportive immune programs (157). Stromal activation markers such as FAP-α have been associated with advanced disease and may be linked to immunosuppressive signaling (157). Preclinical studies demonstrated that CAFs may promote tumor progression through several mechanisms, including CXCL12-mediated migration and chemoresistance, as well as IL-6/JAK2/STAT3- and SOX4-associated feedback loops that support persistence and invasiveness (137, 158). Therapeutic approaches remain largely preclinical.

### 6.4 Regulatory T-cells

Regulatory T cells (Tregs) appear to play a complex role in MF and SS, potentially functioning both as part of the malignant compartment and as contributors to local immunosuppression. In a subset of patients malignant CTCL cells themselves may adopt Treg-like features, including expression of FOXP3, CD25, and CTLA-4, as well as secretion of IL-10 and TGF-β (159). However, this concept remains controversial with other studies demonstrating FOXP3-negativity in the neoplastic infiltrate (160). Non-malignant FOXP3^+^ Tregs have shown stage-dependent dynamics. Increased infiltration has been associated with early-stage disease and, in some cohorts, more favorable outcome, while circulating Tregs in SS appear expanded but functionally heterogeneous (161, 162).

These observations may be therapeutically relevant. Agents such as mogamulizumab and denileukin diftitox may partly reduce immunosuppression through depletion of CCR4+ or CD25+ Tregs in addition to targeting malignant cells (127, 163). Other immunomodulatory strategies, including checkpoint blockade, interferon-α, and ECP, may further reshape the Treg compartment indirectly.

### 6.5 The role of *Staphylococcus aureus*

The increasingly immunosuppressive microenvironment in MF and SS has direct clinical consequences. Patients have been shown to be frequently colonized with *Staphylococcus aureus* and are vulnerable to infectious complications, including fatal sepsis (164). This likely reflects both the Th2-skewed immune milieu and impaired skin barrier function. In parallel, *S. aureus* and its enterotoxins may further disrupt the epithelial barrier by inducing barrier-repressing cytokines (165). Beyond opportunistic colonization, *S. aureus* appears to actively promote disease progression through superantigen-driven crosstalk between malignant and non-malignant T cell, resulting in STAT3- and STAT5-mediated oncogenic signaling (166, 167). Moreover, staphylococcal alpha-toxin may selectively deplete non-malignant T cells while sparing malignant cells, thereby impairing immune surveillance (168). Clinically, antibiotic eradication of *S. aureus* has been associated with reduced in disease activity, decreased malignant T-cell burden in lesional skin, and partial restoration of skin barrier protein expression, although the impact on overall survival remains uncertain (164, 169, 170).

## 7 Immune escape mechanisms

Multiple mechanisms have been implicated in immune evasion in MF and SS, including the dysregulation of adaptive and innate immune checkpoint pathways. Improved understanding of these processes has led to the development of therapeutic strategies targeting immune escape mechanisms to restore anti-tumor immunity. An overview of immune checkpoint-directed therapeutic strategies, their current stage of clinical development, and major limitations is provided in **Table 6**.

**Table 6:** Immune checkpoint–directed therapeutic strategies in Mycosis Fungoides (MF) and Sézary Syndrome (SS).

Target	Therapeutic modality	Repersentative agent(s)	Development status (CTCL)	Major limitations
PD-1	PD-1 blockade	Pembrolizumab	Phase II / NCCN-listed	Tumor flare; hyperprogression risk
PD-1	PD-1 blockade	Tislelizumab	Phase II	Small study size; not FDA-approved
PD-1	PD-1 blockade	Nivolumab	Phase I	Limited CTCL-specific data
PD-1-based combinations	Combination immunotherapy	Pembrolizumab+ Mogamulizumab,Romidepsin, Pralatrexate, Radiotherapy; Nivolumab+ Duvelisib	Phase I–II	Preliminary efficacy; limited cohort sizes
PD-L1	PD-L1 blockade	Durvalumab± Lenalidomide	Phase I/II	Small cohorts; not FDA-approved
PD-L1	PD-L1 blockade	Atezolizumab	Phase I	Limited efficacy and safety data
CTLA-4	Checkpoint modulation	Experimental / indirect (via combination strategies)	Preclinical / early clinical	Malignant T cells themselves express CTLA-4; context-dependent effects; risk of autoimmunity

Overview of immune checkpoint–directed therapeutic strategies, representative agents, current stage of clinical development, and major limitations in mycosis fungoides (MF) and Sézary syndrome (SS). CTCL, cutaneous T-cell lymphoma; CTLA-4, cytotoxic T-lymphocyte-associated protein 4; FDA, U.S. Food and Drug Administration; NCCN, National Comprehensive Cancer Network; PD-1, programmed cell death protein 1; PD-L1, programmed death-ligand 1.

### 7.1 Adaptive checkpoints

Immune checkpoints such as PD-1/PD-L1 and CTLA-4 regulate T-cell activation and are frequently exploited in malignancies to sustain an immunosuppressive microenvironment. In contrast to many solid tumors, checkpoint biology in CTCL is complicated by the fact that malignant CD4^+^ T cells themselves express PD-1, CTLA-4, and other exhaustion-associated markers (149). Preclinical data suggest that these checkpoint pathways suppress effector T-cell function and reinforce Th2-skewed immune polarization, although PD-1 signaling in malignant cells may also exert context-dependent antiproliferative effects (79, 171).

Consistent with this complexity, checkpoint inhibition has shown measurable but variable clinical activity. In the phase II CITN-10 trial, Pembrolizumab achieved an ORR of 38% with generally manageable toxicity (172). Combination approaches, including pembrolizumab plus interferon-γ, appear feasible (173). By contrast, PD-L1 blockade with atezolizumab has shown more modest activity, and early nivolumab data remain limited (174, 175). The most common adverse events associated with atezolizumab were fatigue and infections, with two sepsis-related deaths reported (174).

Checkpoint inhibition in CTCL is further complicated by reports of paradoxic CTCL hyperprogression following PD-1 blockade (176). These observations support a biomarker-guided and combination-based approach, including combinations with anti-CCR4, JAK inhibitors, HDAC inhibitors, or innate checkpoint blockade.

### 7.2 Innate immune checkpoints

Innate immune escape in both MF and SS involves suppression of myeloid effector functions, most prominently through the CD47-SIRPα axis (177). By engaging SIRPα on macrophages, malignant T cells deliver a “don’t eat me” signal that inhibits phagocytosis and supports tumor persistence (178). This provided the rationale for therapeutic interruption of the CD47-SIRPα interaction, either my targeting CD47 on tumor cells or SIRPα on macrophages. In early clinical studies, intralesional administration of the SIRPα–Fc fusion protein ontorpacept showed encouraging activity in relapsed/refractory CTCL, with reductions in tumor burden and circulating Sézary cells (179). However, the broader clinical applicability of CD47-SIRPα-directed therapies remains limited by the ubiquitous expression of CD47 on normal hematopoietic cells, resulting in an “antigen-sink” effect and potential hematologic toxicity (180). In addition, responses appear stronger after intralesional than intravenous administration, suggesting predominantly locoregional activity (179). The absence of predictive biomarkers and potential compensatory immune-evasion mechanisms may limit the efficacy of monotherapy and support combination approaches.

## 8 Fc-mediated effector mechanisms: ADCC and ADCP

Antibody-dependent cellular cytotoxicity (ADCC) and antibody-dependent cellular phagocytosis (ADCP) represent key Fc-mediated effector mechanisms exploited by therapeutic antibodies in CTCL. ADCC is primarily mediated by NK cells via FcγRIIIa (CD16A), whereas ADCP involves macrophage-mediated uptake of opsonized tumor cells (181, 182).

The strongest clinical evidence for ADCC-based therapy in CTCL comes from mogamulizumab, a defucosylated anti-CCR4 antibody that enhances FcγRIIIa binding and NK cell activation. In the phase III MAVORIC trial, it demonstrated superior efficacy to vorinostat in relapsed/refractory CTCL (ORR 28% vs. 5%), with particularly strong activity in the blood compartment and in SS (183). Notably, responses did not clearly correlate with CCR4 expression, possibly reflecting additional effects such as depletion of CCR4^+^ regulatory T cells (183). Combination strategies with mogamulizumab, including ECP, radiation, and immunotherapies, are under investigation (184). Additional agents support the relevance of Fc-mediated mechanisms, including lacutamab, alemtuzumab, and emerging anti-CCR8 antibodies (88, 99, 130).

Despite these advances, Fc-mediated efficacy remains context-dependent. NK-cell dysfunction, lymphopenia, and CD16A shedding can limit effector function, while high MHC class I expression on malignant T cells may inhibit NK activity, particularly in the skin (185, 186). Consistent with this, antibody-based therapies often show greater activity in the blood than in cutaneous lesions. Strategies to enhance Fc-mediated killing include Fc engineering, preservation of CD16A expression, and development of engineered NK cells with enhanced ADCC capacity (187–189).

## 9 Conclusion and future perspectives

This review underlines the biological heterogeneity of MF and SS shaped by the interplay of malignant T-cell-intrinsic alterations, tissue tropism, and microenvironmental immune dysregulation. Rather than reflecting a single defining lesion, MF and SS appear to converge on a limited set of functional programs, including dysregulated signaling, apoptosis resistance, immune escape, and compartment-specific trafficking.

These insights have already shaped therapeutic progress. Agents such as brentuximab vedotin and mogamulizumab have improved outcomes in relapsed or refractory disease, and additional strategies targeting KIR3DL2, CCR8, CD47–SIRPα and survival-associated signaling pathways are expanding the therapeutic landscape. However, responses remain heterogeneous and are determined by disease subtype, tissue compartment, target expression, and local immune context. This is particularly evident for antibody-based therapies, which often show greater activity in blood than in skin, and for checkpoint inhibition, where biologic complexity may limit consistent benefit.

Future progress will likely depend on biologically guided combination strategies rather than further expansion of single-agent therapy alone. Combinations that concurrently target malignant cell-intrinsic survival programs, microenvironmental support, trafficking pathways, and immune escape mechanisms may be particularly relevant. At the same time, more refined biomarker-driven patient stratification will be needed to identify pathway dependence, immune context, and putative therapeutic vulnerabilities.

Several priorities for therapy management emerge from current evidence. These include better integration of compartment-specific biology across skin, blood, and lymph nodes. In addition, prospective validation of emerging targets, and deeper characterization of resistance mechanisms including apoptotic buffering, stromal support and adaptive or innate immune escape have to be considered to optimize therapeutic management. Taken together, these efforts may help move the management of MF and SS toward a more personalized and mechanism-based therapeutic framework.

Overall, although MF and SS remain challenging to treat, the increasing integration of molecular and immune profiling with targeted immunotherapy provides a strong foundation for more precise and durable treatment strategies.
